# Comparison of two approaches for measuring household wealth *via *an asset-based index in rural and peri-urban settings of Hunan province, China

**DOI:** 10.1186/1742-7622-7-7

**Published:** 2010-09-03

**Authors:** Julie Balen, Donald P McManus, Yue-Sheng Li, Zheng-Yuan Zhao, Li-Ping Yuan, Jürg Utzinger, Gail M Williams, Ying Li, Mao-Yuan Ren, Zong-Chuan Liu, Jie Zhou, Giovanna Raso

**Affiliations:** 1 Queensland Institute of Medical Research, P.O. Royal Brisbane Hospital, Brisbane 4029, Queensland, Australia; 2 School of Population Health, University of Queensland, Herston Road, Brisbane 4029, Queensland, Australia; 3 Centre for Non-Traditional Security Studies, S. Rajaratnam School of International Studies, Nanyang Technological University, Nanyang Avenue, Singapore; 4 Department of Global Health and Development, London School of Hygiene and Tropical Medicine, 15-17 Tavistock Place, London WC1 H 9SH, UK; 5 Hunan Institute of Parasitic Diseases, Huabanqiao Road, Yueyang 414000, China; 6 School of Public Health, Central South University, Changsha 410083, China; 7 Department of Epidemiology and Public Health, Swiss Tropical and Public Health Institute, P.O. Box, CH-4002 Basel, Switzerland; 8 University of Basel, P.O. Box, CH-4003 Basel, Switzerland; 9 Département Environnement et Santé, Centre Suisse de Recherches Scientifiques en Côte d'Ivoire, 01 BP 1301, Abidjan 01, Côte d'Ivoire

## Abstract

**Background:**

There are growing concerns regarding inequities in health, with poverty being an important determinant of health as well as a product of health status. Within the People's Republic of China (P.R. China), disparities in socio-economic position are apparent, with the rural-urban gap of particular concern. Our aim was to compare direct and proxy methods of estimating household wealth in a rural and a peri-urban setting of Hunan province, P.R. China.

**Methods:**

We collected data on ownership of household durable assets, housing characteristics, and utility and sanitation variables in two village-wide surveys in Hunan province. We employed principal components analysis (PCA) and principal axis factoring (PAF) to generate household asset-based proxy wealth indices. Households were grouped into quartiles, from 'most wealthy' to 'most poor'. We compared the estimated household wealth for each approach. Asset-based proxy wealth indices were compared to those based on self-reported average annual income and savings at the household level.

**Results:**

Spearman's rank correlation analysis revealed that PCA and PAF yielded similar results, indicating that either approach may be used for estimating household wealth. In both settings investigated, the two indices were significantly associated with self-reported average annual income and combined income and savings, but not with savings alone. However, low correlation coefficients between the proxy and direct measures of wealth indicated that they are not complementary. We found wide disparities in ownership of household durable assets, and utility and sanitation variables, within and between settings.

**Conclusion:**

PCA and PAF yielded almost identical results and generated robust proxy wealth indices and categories. Pooled data from the rural and peri-urban settings highlighted structural differences in wealth, most likely a result of localized urbanization and modernization. Further research is needed to improve measurements of wealth in low-income and transitional country contexts.

## Introduction

Poverty and people's health status are intimately connected, yet the relationship between them is complex and bi-directional [1,2]. On one hand, ill-health may lead to economic poverty [1], or a decrease in expendable income due to high medical bills and/or *via *a direct reduction, or loss, of wages throughout an illness [3]. On the other hand, poor health may result from poverty [1], including an inability to afford adequate nutrition, sanitation, housing, education and healthcare, and poverty-related lifestyle factors that increase disease risk and/or decrease access to medical facilities and services [4,5]. In the People's Republic of China (P.R. China), rapid economic growth and human development over the past three decades has brought over 300 million people out of poverty (arbitrarily defined as living on less than US$ 1 per day) and has vastly improved the overall health status of the population [6]. However, it has also affected the course of income distribution such that disparities in socio-economic position (SEP; for a definition, see Appendix) are currently among the most important social policy issues in the country [7]. Inequalities appear to be widening both across and within different provinces in P.R. China, with the rural-urban gap of particular concern [7]. Since SEP is an important determinant of health, it is conceivable that such disparities will lead to large gaps in health care provision within P.R. China [8]. In order to plan, implement and monitor health programs and other publicly or privately provided services in an equitable way, it is necessary to identify the poor, including individuals or households with low SEP, who might be more vulnerable to poor health outcomes [5].

While SEP can be measured on multiple levels [1], in the past it was mostly determined using an individual's education level, sometimes in combination with their occupation. Currently, approaches for measuring household SEP include 'direct' measures of economic status, including (i) income, (ii) expenditure, and (iii) financial assets (e.g., savings and pensions), and 'proxy' measures (e.g., household durable assets (Appendix), housing characteristics and access to utilities and sanitation) developed from the wealth index originally proposed by Rutstein in the mid-1990 s [9]. Direct measurements can be expensive to collect and may require complex statistical analyses that are beyond the scope of many population health studies [5,10-12]. In developing country settings in particular, large seasonal variability in earnings and a high rate of self-employment, together with potential recall bias and false reporting, may render such data inaccurate or even unreliable [10]. Proxy measures are thought to be more reliable, since they require only data collected using readily available household questionnaires supported by direct observation. A study carried out in southeast Nigeria, however, questioned whether proxy measures are indeed more reliable than direct measurements [11]. From a public health point of view, the proxy wealth index approach is more useful than that of direct measures, since it explains the same, or a greater, amount of the differences between households on a set of health indicators than an income/expenditure index, while requiring far less effort from respondents, interviewers, data processors and analysts [10]. Additionally, proxy measures might be more accurate approximations of SEP, as they measure financial stock ('permanent income') rather than flow ('current income'), and hence are less prone to fluctuation [10,12-14].

Due to the large volume of potentially redundant asset data produced, a data reduction technique known as exploratory factor analysis is often utilized. Exploratory factor analysis evaluates the most meaningful basis to re-express a large, pre-determined set of variables, exploring the relationships between them and filtering out noise to reveal indicators that map most strongly to an underlying latent structure. Two common methods of extracting that structure are principal components analysis (PCA; Appendix) and principal axis factoring (PAF; Appendix), which describe variation among the observed variables *via *a set of derived uncorrelated variables referred to as principal components (PCs) or principal factors (PFs), respectively [15]. Although these two methods often yield similar results, the former is preferred as a method for data reduction, while the latter is widely used for detecting structure within the data. Previously, studies have used either PCA or PAF but comparisons between these two approaches are rare. Based on the inter-relationship between the set of variables, exploratory factor analysis also assigns weights to ownership of the assets. The weights correspond to the factor loadings (eigenvectors; Appendix) of the first derived variable, and are used to generate an index of relative SEP. Using weights derived through exploratory factor analysis may be a more appropriate method of assigning weights to the variables than the more simplistic equal weights method, the complex weighted-by-price-of-item approach or on an ad-hoc basis [16].

Few studies have attempted to verify the extent to which the asset-based index approach is a good proxy for household economic wealth. Concerns include the handling of publicly provided goods and services, and the direct effects of the indicator variables that make up indices, as well as ways of adjusting for household size and age-composition [17,18]. The increasingly widespread use and application of proxy measurements of household economic wealth and SEP, and growing use of exploratory factor analysis, in public health studies calls for further research in this area, particularly in low-income settings and transitional countries.

Here we report the application of exploratory factor analysis to household data that were collected during a survey of parasitic infections in Hunan province, P.R. China. Our aim was to calculate and examine asset-based proxy wealth indices generated by PCA and PAF, and to compare them to other measures of wealth based on purely economic variables, including self-reported annual household income and savings. Results are reported for a rural and a peri-urban (Appendix) setting and aggregated between the two.

## Methods

### Study area and population

The study was carried out in two villages; namely (i) Wuyi, in Hanshou county, southern Dongting Lake area, and (ii) Laogang, in Yueyang county, eastern Dongting Lake area. Both villages are located in Hunan province. The surveys were conducted between November and December 2006. The villages were selected on the basis of previous studies investigating the epidemiology of parasitic infections, including schistosomiasis [19]. Wuyi is situated in a rural area, whereas Laogang is peri-urban, located on the outskirts of Yueyang, the major city in the Dongting Lake region. All individuals from both villages were invited to participate in the study.

### Field procedures

Senior personnel from two local schistosomiasis control stations were involved in co-ordinating the study. Basic demographic information was obtained from a census performed one year previously in both villages. The questionnaire was translated into Mandarin Chinese, back-translated into English, pre-tested in a nearby village and readily adapted to the local setting. It was administered to the heads of households, and included questions on household demographics, the number of wage earners and non-wage earners, annual household income (7 categories: <500; 500-1999; 2000-4999; 5000-9999; 10,000-29,999; 30,000-49,999; ≥50,000 CNY) and savings (6 categories: <500; 500-699; 700-999; 1000-1499; 1500-1999; ≥2000 CNY), the primary and secondary sources of income, ownership of 22 household durable assets (e.g., color TV, washing machine, air conditioner, etc.), 10 housing characteristics (e.g., floor material, wall material, roof material, etc.) and six utility (Appendix) and sanitation variables (e.g., tap water, toilet in house, etc.).

Interviewers were familiar with the local setting and dialect and were acquainted with qualitative methods. The head of each household was invited to respond to the questions; if the household head was absent on the day of interview, the interviewer returned to that residence the following day, for up to a period of 14 days, after which the next of kin was asked to respond.

### Consent and ethical approval

Ethical clearance for this study was obtained from the Medical Ethics Committee of Hunan province and Queensland Institute of Medical Research. Village authorities were informed about the aims and procedure of the study and provided written informed consent. Oral informed consent was obtained from each individual.

### Data management and statistical analysis

#### Data management

Data were double-entered into a bilingual Microsoft^® ^Access 2002 database, cross-checked and subsequently analyzed with SPSS version 16.0 (Illinois, USA).

#### Socio-economic data and asset ownership

Household income and savings data were equivalized to adjust for household needs based upon the number of household members (per-capita) and a combination of number and age (per-adult, defined as individuals aged >16 years). This was done using the median value of each income or savings band and dividing by the number of members or adult members per household. Annual per-capita income and per-adult income, primary source of income, ability to save (yes or no binary variable) and annual per-capita and per-adult savings were then examined using a χ^2 ^test. The Student's *t*-test statistic was used to compare the mean age and mean household size between the two villages. A stepwise multinomial logistic regression analysis with annual per-capita income bands as the dependent variable and ownership of household durable asset as independent binary covariates was used to test the association between household income and asset ownership within each setting separately and for the pooled data from both villages. Covariates were included at a significance level of <0.2. Covariates that were not significantly associated with income were removed in a stepwise backward elimination process. Adjusted odds ratios (OR) and 95% confidence intervals (CI) were computed for associations with p-values <0.05.

#### Construction of asset-based proxy wealth indices using PCA and PAF

A detailed protocol of how we constructed asset-based proxy wealth indices is given in the Additional File. In brief, the binary data on household durable assets, housing characteristics and utility and sanitation variables were organized into a matrix with *m *households as rows (where *m_rural _*= 258 and *m_peri-urban _*= 246) and *n *variables as columns. The initial *n *= 38-item correlation matrices for each setting were examined for internal consistency (Table 1). To enable the matrix to be factorable, only variables with sufficient correlation (φ > |0.3|) with at least three other variables were included in further analyses. If any variable correlated highly (φ > |0.8|) with other variables, only one variable from the group of correlated variables was arbitrarily selected and included in further analyses, to avoid multicollinearity. Factorability of the *m *by *n *matrices was determined using Bartlett's test of sphericity (Appendix) and the Kaiser-Meyer-Olkin (KMO) test (Appendix). Variables were excluded in a stepwise manner until a factorable *m *by *n *correlation matrix with a KMO >0.7 was reached, for each village separately. Diagonal and off-diagonal values of the anti-image correlation matrix (Appendix) were used to assess the sampling adequacy.

**Table 1:** Demographic variables of the respondents in a household-based questionnaire survey in rural (Wuyi village) and peri-urban (Laogang village) settings of Hunan province, China

Demographic variable	Rural (n = 258) (%)	Peri-urban (n = 246) (%)	χ^2^	p-value
Sex				
Male	204 (79.1)	142 (57.7)		
Female	54 (20.9)	104 (42.3)	27.4	< 0.001
Head of household				
Yes	207 (80.2)	167 (67.9)		
No	51 (19.8)	79 (32.1)	10.0	0.002
Age (years)				
Mean (SD)^1^	48.9 (12.3)	51.0 (12.6)		
Household size				
1 individual	17 (6.6)	24 (9.8)	1.7	0.194
2 individuals	101 (39.2)	97 (39.4)	0.0	0.948
3 individuals	71 (27.5)	95 (38.6)	7.0	0.008
≥4 individuals	69 (26.7)	30 (12.2)	16.9	< 0.001
Mean household size (SD)^2^	2.9 (1.3)	2.6 (1.0)		
Number of earners/household				
No earners	5 (1.9)	3 (1.2)	0.4	0.519
1 earner	30 (11.6)	77 (31.3)	29.1	< 0.001
2 earners	190 (73.7)	146 (59.4)	11.6	0.001
3 earners	16 (6.2)	15 (6.1)	0.0	0.961
>4 earners	17 (6.6)	5 (2.0)	6.3	0.012
Mean household size per number of earner (SD)^3^	1.5 (0.7)	1.6 (0.8)		

^1 ^*t*-test statistic = -1.9, d.f. = 492, p = 0.062

^2 ^*t*-test statistic = 3.0, d.f. = 502, p = 0.003

^3 ^*t*-test statistic = -1.9 d.f. = 494, p = 0.054

Next, components and factors were extracted from each of the final two correlation matrices using PCA and PAF, respectively. Components and factors, respectively, were extracted without and with rotation (Appendix) and the best method was selected according to the maximum squared factor loadings and the relative simplicity of the model. In each case, eigenvalues >1 (Appendix), examination of the scree plots and the cumulative proportion of variance explained by each component or factor were taken as criteria for extraction. For simplicity, a cut-off eigenvector > |0.3| was used to signify component or factor loadings of interest and, where variables loaded equally on more than one component or factor, the Cronbach's coefficient α (Appendix) was used to select the component or factor on which to place the variable.

The PC and PF loadings were used to compute standardized indices of relative household wealth within each village, according to the following equation:

$$A_{i}\;=\;{\hat{\gamma }}_{1}\alpha_{i1}\;+\;\ldots \;+{\hat{\gamma }}_{k}\alpha_{ik}$$

where $\alpha_{ik}\;=\;\left(x_{ik}-{\bar{x}}_{k}\right)/S_{k}$

such that *A_i _*is the standardized asset index score per household *i*, the *_k_*s are the factor loadings or weights of each asset *k*, estimated by either PCA or PAF, and the *α_ik_*s are the standardized values of asset *k *for household *i *(i.e., *x_ik _*is the ownership of asset *k *by household *i*, where 0 represents not owning the asset and 1 represents owning the asset, and ${\bar{x}}_{k}$and *s_k _*are the sample mean and standard deviation (SD) of asset *k *for all households).

The association between the PCA- and PAF-based proxy wealth indices was estimated by the Spearman's rank correlation coefficient (Appendix). Based on the overall small sample size of our study, we chose to divide each index into quartiles, rather than the standard quintiles or tertiles, representing: (i) most poor (MP), (ii) below average (BA), (iii) above average (AA), and (iv) most wealthy (MW) households.

#### Proxy wealth indices and self-reported income and savings

Corresponding wealth quartiles were also generated based on annual household per-capita income and on a combination of household income and savings, as follows: (i) high income (≥4000 CNY per person per year) with savings, (ii) high income without savings, (iii) low income (< 4000 CNY per person per year) with savings, and (iv) low income without savings. Households' categorical position for each respective index was assessed by a Kappa agreement, using the following cut-offs: 0, no agreement; 0.01-0.2, poor agreement; 0.21-0.4, fair agreement; 0.41-0.6, moderate agreement; 0.61-0.8, substantial agreement; 0.81-1, almost perfect agreement [20]. Households that were re-ranked into different quartiles were examined in further detail. Mean scores per category were examined by means of Kruskal-Wallis (Appendix) analyses and a ratio of MW to MP was calculated. This entire process was then repeated for the pooled data from both villages (*m_total _*= 504).

## Results

### Study compliance and operational results

From a total of 646 households in both villages, 504 (78.0%) had complete datasets. This corresponded to 258/294 (87.8%) in the rural setting and 246/352 (69.9%) in the peri-urban setting. Demographic variables are summarized in Table 1.

### Comparison of income, savings, and possession of assets

Table 2 shows annual household per-capita income and per-adult income, the primary source of income and the ability to save based on the primary source of income, for both settings. Annual household per-capita income was significantly associated with village setting (χ^2 ^test p <0.001), as was annual household per-adult income (χ^2 ^test p <0.001). The primary source of household income was also associated with village setting (χ^2 ^test p <0.001). Overall, 156 (31.0%) households reported the ability to save money; however this was more frequent in the rural setting (106 or 41.1% *vs*. peri-urban: 50 or 20.3%; χ^2 ^test p <0.001). Within both villages, saving was positively associated with annual household per-capita income (χ^2 ^test p <0.001 and χ^2 ^test p <0.001) and varied significantly according to primary source of income (χ^2 ^test p = 0.040 and χ^2 ^test p = 0.027) for Wuyi and Laogang, respectively. In Wuyi, younger household heads were more likely to save than their older counterparts (χ^2 ^test p = 0.006), while there was no significant difference in Laogang. Within both settings, the amount of money saved per capita was also positively associated with annual household per-capita income (χ^2 ^test p <0.001 and χ^2 ^test p = 0.001), but not with the primary source of household income or with age for rural and peri-urban settings.

**Table 2:** Self-reported annual household income and savings in rural (Wuyi village) and peri-urban (Laogang village) settings of Hunan province, China

Income and savings	Rural (n = 258) (%)	Peri-urban (n = 246) (%)	χ^2^	p-value
*Annual household per-capita income *(CNY) *				
<1000	24 (9.3)	4 (1.6)	14.1	< 0.001
1000-1999	55 (21.3)	26 (10.6)	10.8	0.001
2000-3499	19 (7.4)	52 (21.1)	19.7	< 0.001
3500-4999	66 (25.6)	65 (26.4)	0.0	0.830
5000-6999	37 (14.3)	46 (18.7)	2.1	0.147
7000-8999	40 (15.5)	47 (19.1)	0.9	0.341
≥9000	17 (6.6)	6 (2.5)	3.6	0.058
*Primary source of household income*				
Farming	33 (12.8)	33 (13.4)	0.0	0.836
Fishing	149 (57.7)	8 (3.3)	11.7	< 0.001
Farming and fishing	38 (14.7)	0	39.2	< 0.001
Vegetable crops/animal rearing	10 (3.9)	3 (1.2)	3.5	0.060
Small business	8 (3.1)	27 (11.0)	12.0	0.001
Other	20 (7.8)	175 (71.1)	12.1	< 0.001
*Ability to save*				
Overall	106 (41.1)	50 (20.3)	28.5	< 0.001
Farmers	16 (48.5)	5 (15.2)	3.5	0.059
Fishermen	50 (33.6)	1 (12.5)	n.a.	n.a.
Farmers and fishermen	21 (55.3)	0	n.a.	n.a.
Animal rearers	5 (71.4)	0	n.a.	n.a.
Businessmen	6 (75.0)	12 (44.4)	3.4	0.061
Other	8 (40.0)	32 (18.3)	14.0	< 0.001

n.a., not applicable; * Exchange rate CNY 1 ≈ US$ 0.16 at the time of survey (November/December 2006).

Table 3 shows the complete list of household durable assets, housing characteristics and utility and sanitation variables for both settings. Item ownership varied between and within villages. For example, all 246 peri-urban households but only 5 (1.9%) rural households had tap water in the house. While 229 (88.8%) rural households owned animals, the respective number and percentage was 46 (18.7%) among peri-urban households.

**Table 3:** Prevalence of ownership of household durable assets, housing characteristics and utility and sanitation variables in rural (Wuyi village) and peri-urban (Laogang village) settings of Hunan province, China

*Variable*	Rural (n = 258)	Peri-urban (n = 246)	χ^2^	p-value
**Household durable assets**				
1 Own house	236 (91.5)	209 (85.0)	9.5	0.002
2 Own land	216 (83.7)	55 (22.4)	212.9	< 0.001
3 Own animals	229 (88.8)	46 (18.7)	253.5	< 0.001
4 Gas rice cooker	134 (51.9)	241 (98.0)	139.1	< 0.001
5 Microwave	215 (83.3)	227 (92.3)	8.8	0.003
6 Black/white TV	22 (8.5)	10 (4.1)	4.4	0.037
7 Color TV	236 (91.5)	228 (92.7)	0.1	0.720
8 VCR	136 (52.7)	101 (41.6)	6.5	0.011
9 Satellite dish	82 (31.8)	6 (2.4)	75.7	< 0.001
10 Phone line	134 (51.9)	157 (63.8)	7.1	0.008
11 Mobile phone	82 (31.8)	142 (57.7)	33.9	< 0.001
12 Bicycle	161 (62.4)	18 (7.3)	167.9	< 0.001
13 Motorbike	81 (31.4)	18 (7.3)	46.6	< 0.001
14 Electric fan	246 (95.3)	238 (96.7)	0.2	0.694
15 Air conditioner	9 (3.5)	62 (25.2)	48.8	< 0.001
16 Fridge	50 (19.4)	138 (56.1)	72.1	< 0.001
17 Washing machine	124 (48.1)	172 (69.9)	23.9	< 0.001
18 Simple tractor	4 (1.6)	1 (0.4)	1.7	0.194
19 Expensive tractor	8 (3.1)	1 (0.4)	6.3	0.022
20 Truck	1 (0.4)	2 (0.8)	0.4	0.537
21 Boat	106 (41.1)	24 (9.8)	65.6	< 0.001
22 Car	1 (0.4)	7 (2.8)	4.8	0.028
**Housing characteristics**				
23 Weak brick walls	233 (90.3)	134 (54.5)	81.7	< 0.001
24 Strong brick walls	1 (0.4)	112 (45.5)	147.5	< 0.001
25 Wooden walls	22 (8.5)	0	21.9	< 0.001
26 Weak brick roof	169 (65.5)	40 (16.3)	125.8	< 0.001
27 Strong brick roof	86 (33.3)	206 (83.7)	131.3	< 0.001
28 Wooden roof	1 (0.4)	0	1.0	0.328
29 Mud floor	28 (10.9)	3 (1.2)	20.2	< 0.001
30 Cement floor	155 (60.1)	68 (27.6)	53.7	< 0.001
31 Porcelain floor	57 (22.1)	174 (70.7)	120.0	< 0.001
32 Wooden floor	16 (6.2)	1 (0.4)	13.0	< 0.001
**Utility and sanitation**				
33 Tap water	5 (1.9)	246 (100.0)	483.4	< 0.001
34 Hand pump water	254 (98.4)	1 (0.4)	487.2	< 0.001
35 Flushable toilet	96 (37.2)	215 (87.4)	133.4	< 0.001
36 Medicine at home	12 (4.7)	95 (38.6)	86.6	< 0.001
37 Soap	256 (99.2)	242 (98.4)	1.1	0.293
38 Over-crowding	32 (12.4)	16 (6.5)	5.1	0.024

Table 4 summarizes all significant associations between annual per-capita household income and ownership of household durable assets across pooled data from both rural and peri-urban settings, with the model accounting for 48.6% of variation in the data.

**Table 4:** Significant associations between annual per-capita household income and ownership of household durable assets, as assessed by a stepwise multinomial logistic regression analysis.

*Variable*	Annual household per-capita income (CNY) *(n = 504)													
	
	< 1000		1000-1999		2000-3499		3500-4999		5000-6999		7000-8999		> 9000	
	
	OR (95% CI)	p-value	OR (95% CI)	p-value	OR (95% CI)	p-value	OR (95% CI)	p-value	OR (95% CI)	p-value	OR (95% CI)	p-value	OR (95% CI)	p-value
4 Gas rice cooker	2.1 (0.4-4.2)	0.120	2.2 (0.4-5.4)	0.115	2.0 (0.2-4.8)	0.121	2.2 (0.6-7.1)	0.115	4.4 (2.0-6.1)	0.037	2.9 (0.8-3.8)	0.110	3.0 (0.6-5.1)	0.111
7 Color TV	2.8 (0.4-6.7)	0.087	3.8 (2.1-5.6)	0.049	8.5 (6.7-12.0)	0.004	2.9 (0.8-4.0)	0.087	4.9 (2.8-6.3)	0.027	3.1 (0.9-4.9)	0.053	3.6 (1.8-4.5)	0.050
8 VCR	2.2 (0.4-3.7)	0.115	1.0 (0.5-5.2)	0.180	0.6 (0.1-2.5)	1.240	0.6 (0.1-2.1)	1.240	2.5 (0.7-4.3)	0.113	3.6 (1.0-7.7)	0.051	4.0 (1.5-5.8)	0.047
9 Satellite dish	4.4 (3.0-6.1)	0.037	4.3 (2.9-6.6)	0.039	4.9 (3.1-6.0)	0.028	4.0 (2.2-7.1)	0.050	4.3 (2.5-7.4)	0.039	4.4 (2.1-6.8)	0.038	4.8 (2.1-7.6)	0.028
11 Mobile phone	4.1 (3.2-5.6)	0.044	4.0 (3.1-6.2)	0.047	7.3 (4.3-12.9)	0.007	3.5 (3.2-5.3)	0.061	7.4 (4.0-9.8)	0.006	3.2 (2.2-6.8)	0.048	3.5 (2.5-6.9)	0.060
12 Bicycle	6.2 (3.3-9.6)	0.012	6.4 (4.2-8.7)	0.011	6.2 (4.6-9.8)	0.012	8.5 (5.5-12.1)	0.004	7.3 (4.1-12.2)	0.007	3.3 (0.9-5.7)	0.069	2.9 (0.6-3.1)	0.110
14 Electric fan	3.0 (0.6-5.7)	0.110	2.9 (0.4-4.8)	0.110	4.5 (3.9-8.1)	0.034	2.8 (0.6-3.1)	0.111	4.0 (1.4-7.3)	0.047	4.6 (2.0-7.1)	0.030	4.2 (3.3-7.2)	0.041
15 Air conditioner	0.1 (0.2-3.6)	0.839	0.3 (0.0-1.4)	0.550	2.3 (0.6-4.7)	0.129	4.6 (2.8-7.6)	0.033	4.2 (3.3-6.9)	0.040	3.9 (1.5-6.3)	0.049	2.7 (2.0-4.0)	0.112
17 Washing machine	1.4 (0.2-2.3)	0.245	2.9 (0.3-3.3)	0.110	2.0 (0.8-4.4)	0.121	3.1 (1.2-5.6)	0.045	1.4 (0.3-2.2)	0.245	1.6 (0.0-2.6)	0.204	0.3 (1.2-4.9)	0.550

Adjusted odds ratios (OR) including 95% confidence intervals (CI) shown for pooled data from both rural (Wuyi village) and peri-urban (Laogang village) settings of Hunan province, China

The model accounts for 48.6% of variation in the data; * Exchange rate CNY 1 ≈ US$ 0.16 at the time of survey (November/December 2006).

### Comparison of asset-based indices constructed using PCA and PAF

Examination of the initial correlation matrix for both settings identified inter-item φ correlations >0.8, excluding numerous variables from further analyses. The final correlation matrix consisted of 15 variables for Wuyi and 11 variables for Laogang, and 14 variables for the pooled data (Table 5).

**Table 5:** Principal components (PCs) or principal factors (PFs) extracted by principal components analysis (PCA) and principal axis factoring (PAF), showing component or factor loadings.

***Variable*** **(% variance accounted for)**	**Rural (Wuyi)**				**Peri-urban (Laogang)**		
		
	**PC 1, PF 1** **(24.3%)**	**PC 2, PF 2** **(11.0%)**	**PC 3, PF 3** **(9.4%)**	**PC 4, PF 4** **(8.9%)**	**PC 1, PF 1** **(27.8%)**	**PC 2, PF 2** **(16.5%)**	**PC 3, PF 3** **(12.7%)**
	
2 Own land			- 0.421, n.a.	**0.564**, 0.436		0.574, -0.408	
3 Own animals			- 0.469, n.a.	**0.493**, n.a.	n.a.	n.a.	n.a.
4 Gas rice cooker	0.638, 0.573				n.a.	n.a.	n.a.
5 Microwave	0.464, 0.388				n.a.	n.a.	n.a.
8 VCR	**0.465**, 0.405	0.444, n.a.			0.702, 0.613		
9 Satellite dish	0.527, 0.483				n.a.	n.a.	n.a.
10 Phone line	n.a.	n.a.	n.a.	n.a.		-0.736, 0.527	
11 Mobile phone	0.611, 0.553				0.668, 0.585		
13 Motorbike	0.595, 0.525				n.a.	n.a.	n.a.
15 Air conditioner	n.a.	n.a.	n.a.	n.a.	0.585, 0.500		
16 Fridge	0.444, 0.373				0.661, 0.614		
17 Washing machine	**0.580**, 0.533	0.430, n.a.			0.734, 0.668		
21 Boat				0.669, 0.429			
27 Weak brick roof	- 0.564,-0.554				n.a.	n.a.	n.a.
28 Strong brick roof	n.a.	n.a.	n.a.	n.a.		0.827, 0.849	
31 Porcelain floor	0.605, **0.601**	n.a., -0.482			0.650**, 0.647**		n.a., -0.500
35 Flushable toilet	0.694, 0.676				0.561, 0.512		
36 Medicines at home	n.a.	n.a.	n.a.	n.a.			0.677, 0.433
38 Over-crowding			0.678, 0.453		n.a.	n.a.	n.a.

Values shown are for rural (Wuyi village) (left) and peri-urban (Laogang village) (right) settings, Hunan province, China*

n.a, not applicable; ^1 ^Factor loadings reported only if they exceed the cut-off eigenvector of |0.3|;* Rotation method: no rotation used to maximize the squared loadings of the columns.

**NB **Indices were created using only the first component or factor. Remaining components or factors are shown for clarification purposes. Where variables loaded on more than one component or factor, the one selected is shown in **bold**. This was done based on the co-efficient α score.

Bartlett's test of sphericity was significant in both settings (rural: χ^2 ^test p <0.001 and peri-urban: χ^2 ^test p <0.001) and for the pooled data (χ^2 ^test p <0.001), and the respective KMO statistics were 0.788, 0.726 and 0.863. The anti-image correlation matrix measures of sampling adequacy were above 0.636 and the off-diagonal values were below |0.345| for each setting and pooled data. Cronbach's coefficient α for the 15-item scale was 0.652 in Wuyi, 0.615 for the 11-item scale in Laogang, and 0.667 for the 14-item scale pooled data.

PCA and PAF revealed four components or factors with eigenvalues >1.0 in the rural setting and three in the peri-urban setting. In each case the first component or factor comprised of several heavily loaded variables (eigenvectors >0.3) and accounted for 24.3% and 27.8% of the variation in the data from Wuyi and Laogang, respectively, while the remaining components or factors had fewer variables and explained a smaller proportion of the variation (Table 5). For the pooled data, three components or factors had eigenvalues >1.0 and the first component or factor accounted for 33.9% of the variation in the data. The un-rotated extraction method was selected for PCA and PAF in both settings and for the pooled data, as rotation did not add measurably to the simplicity or fit of each of the models. The relative magnitude and direction of the weights in the PCA and PAF models are consistent within settings (Table 5) and across pooled data (data not shown).

**Figure 1:**
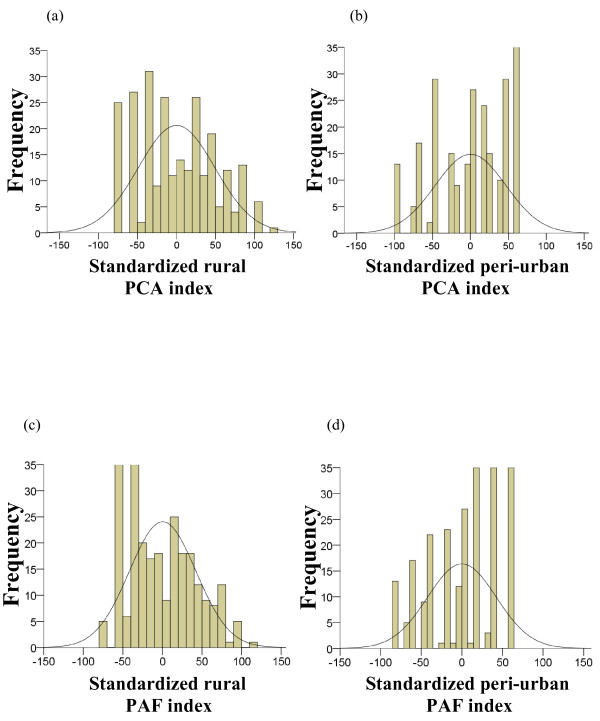
**Distribution of the standardized asset-based proxy wealth index scores created using exploratory factor analysis with the principal components analysis (PCA) extraction method (a, c) and the principal axis factoring (PAF) extraction method (b, d) in rural (Wuyi village) and peri-urban (Laogang village) settings, Hunan province, P.R. China**.

**Figure 2:**
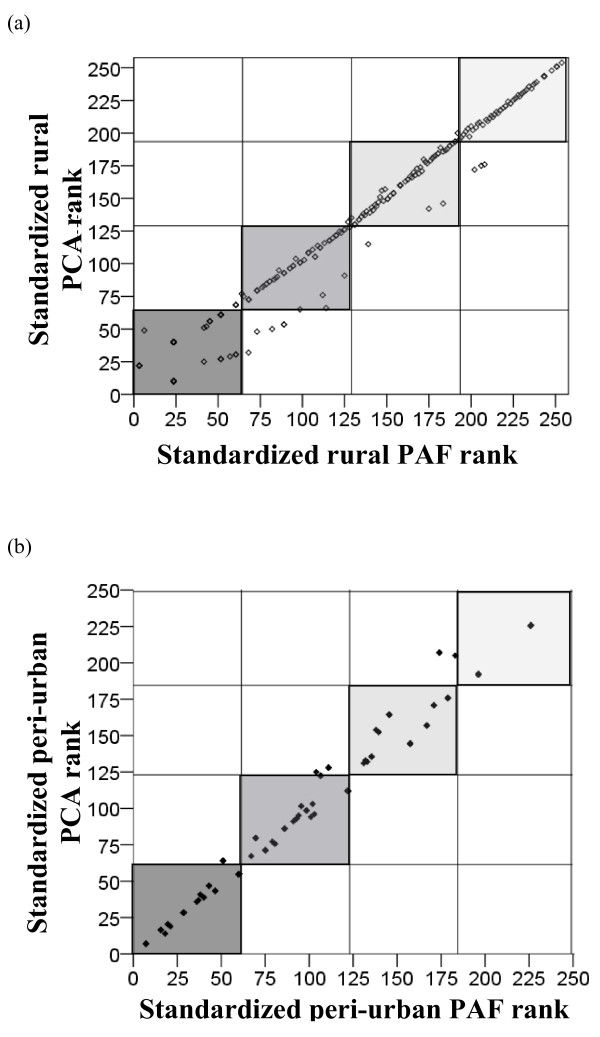
**Correlation of the standardized asset-based proxy wealth index scores created using exploratory factor analysis with the principal components analysis (PCA) and the principal axis factoring (PAF) extraction methods in (a) rural (Wuyi village) and (b) peri-urban (Laogang village) settings of Hunan province, P.R. China**. Lines vertical to the axes define the respective wealth quartiles of each index for rural (dashed) and peri-urban (dotted) settings, respectively.

For both settings, standardized indices of relative wealth were created using heavily loaded variables of the first PC, or the first PF, with variables weighted according to their eigenvector, as in the equation. All four indices showed evidence of clumping and truncation (Figure 1). The PCA and PAF indices correlated well with each other within each village (for both settings Spearman's rho = 0.99, p <0.001). The Kappa agreement was found to be almost perfect, with values of 0.91 and 0.81 for Wuyi and Laogang, respectively. In Wuyi, 17 (6.6%) households were in different quartiles according to factor extraction method, while this was the case for 35 (14.2%) households in Laogang (Figure 2).

### Comparison of proxy wealth indices with self-reported income and savings

**Figure 3:**
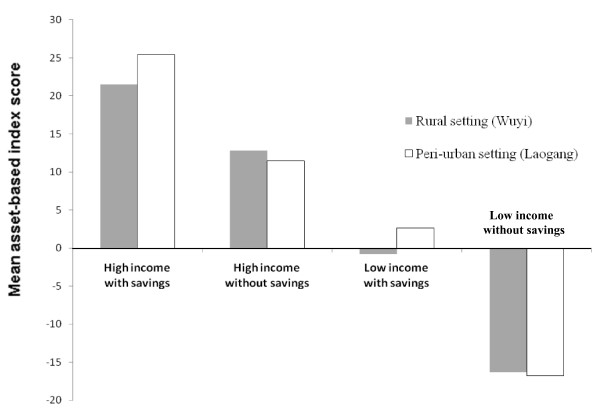
**Mean asset-based index scores, derived either by principal components analysis (PCA), according to income and savings categories**. Values shown are for rural (Wuyi village) (filled) and peri-urban (Laogang village) (blank) settings, Hunan province, P.R. China.

Both PCA and PAF indices showed a weak, but significant, positive correlation with annual household per-capita income (Spearman's rho = 0.27, p <0.001 for PCA and Spearman's rho = 0.26, p <0.001 for PAF), with annual household per-adult income (Spearman's rho = 0.30, p <0.001 for PCA and Spearman's rho = 0.29, p <0.001 for PAF) and with annual household per-capita savings (Spearman's rho = 0.16, p = 0.016 for PCA and Spearman's rho = 0.16, p = 0.017 for PAF) in the rural setting. Similarly, in the peri-urban setting both indices were weakly, but significantly, correlated with annual household income (per-capita income Spearman's rho = 0.27, p <0.001 for PCA and Spearman's rho = 0.28, p <0.001 for PAF and per-adult income Spearman's rho = 0.36, p <0.001 for PCA and Spearman's rho = 0.37, p <0.001 for PAF) and with annual household per-capita savings (Spearman's rho = 0.26, p = <0.001 for PCA and Spearman's rho = 0.27, p <0.001 for PAF). Mean asset-based index scores, derived either by PCA or PAF, were significantly different between combined income and savings categories in both settings (PCA rural score (i) high income with savings: 21.5, (ii) high income without savings: 12.8, (iii) low income with savings: -8.0, and (iv) low income without savings: -16.3; PCA peri-urban score (i) high income with savings: 25.4, (ii) high income without savings: 11.5, (iii) low income with savings: 2.6, and (iv) low income without savings: -16.8; Kruskal-Wallis = 27.6, degrees of freedom (d.f.) = 3, p <0.001 for the rural setting and Kruskal-Wallis = 32.0, d.f. = 3, p <0.001 for the peri-urban setting) (Figure 3).

We found wide disparities among the asset-based proxy wealth quartiles in mean annual household per-capita income and per-adult income. To illustrate this issue, using the PCA extraction method, we found highly significant Kruskal-Wallis test results both for rural and peri-urban settings (annual household per-capita income in rural setting Kruskal-Wallis = 14.7, d.f. = 3, p = 0.002 and peri-urban setting Kruskal-Wallis = 21.0, d.f. = 3, p <0.001 and for per-adult income in rural setting Kruskal-Wallis = 23.7, d.f. = 3, p = 0.001 and peri-urban setting Kruskal-Wallis = 35.1, d.f. = 3, p <0.001). Similarly, we found disparities among wealth quartiles in a household's ability to save for both settings (χ^2 ^test p = 0.014 and χ^2 ^test p <0.001 for rural and peri-urban settings, respectively) (Table 6, rural setting only). This pattern was also confirmed when comparing mean annual household per-capita savings among wealth quartiles for the peri-urban setting (Kruskal-Wallis = 17.3, d.f. = 3, p <0.001) but not for the rural setting (Kruskal-Wallis = 6.9, d.f. = 3, p = 0.077). Disparities in a combination of annual household income and saving were also apparent between MW and MP quartiles, in both settings (Table 6, rural setting only).

**Table 6:** The relationship between the proxy wealth index generated using principal components analysis (PCA) and income and savings, among households in a rural (Wuyi village) setting, Hunan province, China

*Variable*	Wealth quartile*						
				
	Most poor (n = 64)	Below average (n = 66)	Above average (n = 64)	Most wealthy (n = 64)	MW:MP**	χ^2^	p-value
Ability to save money	15 (23.4%)	23 (34.8%)	29 (45.3%)	39 (60.9%)	2.6	10.6	0.014
Low income without savings	34 (53.1%)	35 (53.0%)	20 (31.3%)	14 (21.9%)	0.4	19.6	< 0.001
Low income with savings	5 (7.8%)	8 (12.1%)	6 (9.4%)	10 (15.6%)	2.0	1.9	0.588
High income without savings	15 (23.4%)	12 (18.2%)	15 (23.4%)	11 (17.2%)	0.7	4.1	0.247
High income with savings	10 (15.6%)	11 (16.7%)	23 (35. 9%)	29 (45.3%)	2.9	20.1	< 0.001

* n = Number of households in each category; ** = Most wealthy to most poor ratio

**Figure 4:**
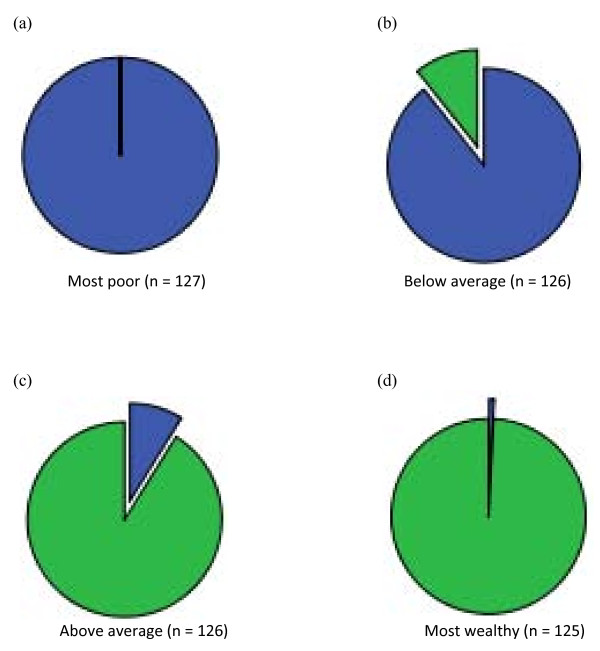
**Proportion of households within each proxy wealth quartile that are in the rural (Wuyi village) (blue) and peri-urban (Laogang village) (green) settings of Hunan province, P.R. China, respectively**. Quartiles represent (a) most poor (n = 127); (b) below average (n = 126); (c) above average (n = 126); and (d) most wealthy (n = 125) categories for pooled data from both villages.

When the analyses were repeated for pooled data, we found that households from each setting were highly unequally distributed among the proxy wealth quartiles (Figure 4). Both PCA- and PAF-based indices showed weak, but significant, positive correlations with annual household per-capita income (Spearman's rho = 0.27, p <0.001 for PCA and Spearman's rho = 0.28, p <0.001 for PAF), per-adult income (Spearman's rho = 0.21, p <0.001 for PCA and Spearman's rho = 0.22, p <0.001 for PAF) and per-capita savings (Spearman's rho = 0.26, p <0.001 for PCA and Spearman's rho = 0.27, p <0.001 for PAF). Kappa agreements of the PCA and PAF indices with the index based on per-capita income were poor (0.12 and 0.13, respectively). Wide disparities in household durable assets, housing characteristics, utilities and sanitation were clear among the four proxy wealth categories. Disparities in a combination of annual household income and saving were also apparent between MW and MP quartiles (Table 7).

**Table 7:** The relationship between the proxy wealth index generated using principal components analysis (PCA) and income and savings, among households in rural (Wuyi village) and peri-urban (Laogan village) settings, Hunan province, China

*Variable*	Wealth quartile*						
				
	Most poor (n = 127)	Below average (n = 126)	Above average (n = 126)	Most wealthy (n = 125)	MW:MP**	χ^2^	p-value
Ability to save money	43 (33.9%)	51 (40.5%)	29 (23.0%)	33 (26.4%)	0.8	15.2	0.002
Low income without savings	59 (46.4%)	44 (34.9%)	66 (52.4%)	54 (43.2%)	0.9	10.8	0.013
Low income with savings	19 (15.0%)	11 (8.7%)	9 (7.1%)	5 (4.0%)	0.3	15.2	0.002
High income without savings	25 (19.7%)	31 (24.6%)	31 (24.6%)	38 (30.4%)	1.5	15.0	0.003
High income with savings	24 (18.9%)	40 (31.8%)	20 (15.9%)	28 (22.4%)	1.2	11.1	0.011

* n = Number of households in each category; ** = Most wealthy to most poor ratio

## Discussion

This study contributes methodologically and analytically to research into measurements of wealth and SEP in a country undergoing rapid social, economic, demographic and health transitions [21]. Using household-level data collected with a pre-tested and standardized questionnaire in a rural and a peri-urban setting in Hunan province, P.R. China, we examined asset-based proxy wealth measurements constructed by two common exploratory factor analysis approaches. Our results confirm that, although they have different underlying theoretical assumptions, both PCA and PAF are equally effective statistical techniques in evaluating relative wealth among households. Consistent with the proxy wealth indices derived in the Demographic and Health Surveys (DHS) [9], we selected the first un-rotated component/factor, which accounted for 24.3% (rural) and 27.8% (peri-urban) of the overall variation in the data. Proxy wealth index scores were significantly associated with wealth quartiles based on a household's self-reported annual income, and a combination of income and savings, but not savings alone. We found large discrepancies between MW and MP households within and between the two study villages. However, further analyses of pooled results suggest that when combining data from the two settings, these differences may be structural, owing more to urbanization, modernization and accessibility of goods and services, rather than wealth *per se*. This may be particularly true for P.R. China, which is undergoing a long-term, yet spatially heterogenous, period of industrialization and development [22-24].

Salaries in the rural setting were frequently at the low (e.g., CNY <2000) and the high (e.g., CNY >7000) ends of the spectrum, while those in the peri-urban setting seemed clustered in the middle. This is possibly explained by reporting bias, as many of the peri-urban respondents were the next of kin and not the household head, or, by unaccounted externalities (e.g., government policies) imposing a spatial correlation on household income [25]. As in most of rural P.R. China, household income was predominantly (in the case of 220, or 85.3%, households) sourced from fishing and/or farming activities [26]. However, in the peri-urban setting our questionnaire survey failed to capture the most common source of primary income (175, or 71.1%, peri-urban household respondents reported 'other' primary source of income), although anecdotal evidence suggests that these are mainly remittances from non-resident household members and occasionally government payments or basic pension schemes. Saving was more commonly reported in the rural setting which, assuming no reporting bias, is likely a result of less secure employment, and hence greater income uncertainty [27], and a weakened social security system bringing about high user charges for public services [28]. We found that age was an important factor in saving patterns of rural households, implying that younger households smooth consumption, perhaps in order to invest so that their living standards can be enhanced in the future. Furthermore, stronger social networks in the rural setting may impact on decision making behavior such as household expenditure patterns, while costs of basic needs may also be substantially lower in rural areas [29,30].

Though proxy measures of wealth are welcome tools in international health research [15], the construction of indices based on exploratory factor analysis has been criticized for being subjective and unstandardized [12,31]. Conversely, several studies have reported that the asset-based index is a more accurate indicator of long-term wealth than income and consumption data [14,15]. Nonetheless, the reliability of the asset-based index has also been questioned by some authors [11]. Indeed, using binary data, such as ownership of a particular asset, may violate underlying assumptions that the measured variables are related in a linear fashion to the underlying latent constructs (i.e., wealth). Our results confirm those in other settings [15,32], indicating internal consistency and robustness in both methods, particularly for higher ranking households. While household income showed a significant association with ownership of numerous household durable assets, the correlation between the asset-based proxy wealth indices and the direct measures of wealth was low. The proxy wealth models explained a higher proportion of data in the peri-urban setting than the rural setting (27.8% *vs*. 24.3% for PCA), which may add strength to the concern that an asset-based index is a more 'appropriate' measure of wealth in urban areas compared with rural areas [18,31]. To increase these percentages, other data analysis tools such as the modified hierarchical ordered probit (HOPIT) model [33] and multiple correspondence analysis (MCA) [34] may be used to weight the indicators and should be explored further in subsequent studies.

Questions remain regarding the choice and number of variables to be included, although it has been suggested that the data should comprise of 10-15 subjects per variable [35]. With 15 and 11 variables in the rural and peri-urban villages, respectively, our sample size of 246-258 households was satisfactory. Sampling adequacy was further confirmed by the KMO measure (KMO >0.7 is said to be 'meritious'), and by Bartlett's test of sphericity, which indicated that the correlation matrices were not identity matrices, and hence the factor model was appropriate. Retaining only components or factors with eigenvalues >1.0 ensured that they explained at least as much variance in the data as one measured variable, since the variance accounted for by each of the components is its associated eigenvalue. However, Cronbach's coefficient α was just below 0.7 for each setting, indicating that up to 50% of the variance in the items may be attributable to measurement error. Similar to other studies, we found that the first PC and the first PF only explained a low percentage of variation in the data (20-30%). This finding suggests that, while the derived indices do provide a proxy measure of wealth, it is estimated with a considerable level of inaccuracy [31,32,36]. Although inclusion of the remaining components or factors helps explain some of the remaining variation, it is unclear if, and how, this should be done [37].

Consistent with findings from other studies, both PCA and PAF showed signs of clumping and truncation, hindering their ability to accurately classify wealth quartile borderline households, although this was less obvious in the rural data [18,32,34] (see Figure 1). Clumping may be a statistical phenomenon caused by a lack of input variables that can adequately distinguish between households of a similar economic status [18], or it may be a product of social and economic homogeneity stemming from half a century of socialist rule in P.R. China [27,31]. In the peri-urban setting, including ownership of computers and household Internet service may have helped further differentiate between the AA and MW. Differentiating between the age, price, condition and quantity of specific assets may reduce the effect of clumping and/or truncation and should be explored in greater detail, although results from previous studies imply that this information may not add to the accuracy or robustness of the index [14,38]. Furthermore, in our study the few households which were re-classified into different quartiles according to the factor extraction method employed only moved to immediately adjacent quartiles.

Notably, our asset-based proxy wealth index includes utility and sanitation variables, which can have direct effects on health, hence making it difficult to separate out indirect effects on health, *via *improved living conditions, from direct ones. Furthermore, a distinction should be made between variables that may be *determinants *of wealth, such as means of production, communication or transport, and those that are purely *indicators *of wealth, such as certain leisure goods [18]. Where quantifying the extent of inequality is the major goal, the concentration index and its associated concentration curve may be used [39,40]. Alternative approaches to measuring wealth, for example participatory wealth ranking (PWR), may be borrowed from development studies or from econometrics, potentially providing new insight for public health researchers [41].

An important drawback of the household survey method employed in our study is that the population sample did not include migrant populations, who tend to be the most poor and socially disadvantaged households in society [42], or information on informal remittances from temporary migrating household members [31]. Furthermore, the census data employed were obtained one year before our survey, and may have become inaccurate in the fast-changing living environment of contemporary P.R. China. Finally, compliance in the peri-urban setting was considerably lower than in the rural setting (70% *vs*. 88%) and no further information was available on non-compliant individuals for comparison [43]. Including the migrant population may have significantly altered the patterns emerging from our aggregated data and the apparently wide systemic rural to peri-urban gap [17].

While it is beyond the scope of this paper to comprehensively explore the factors behind the disparities both within and between settings, we call upon further research into the complex interactions between these and other assets such as human capital, public capital and land assets [44,45]. This would help to establish the driving forces of the observed differences between direct and proxy measures of wealth and to further examine how these differences impact on health service utilization, research and health policy [44,45]. Improved living conditions and diminished inequality gaps are not only important as distal and proximal determinants of health, but are also vital factors for national and regional socio-political stability [29]. Closing the rural to urban gap in particular is currently a top policy priority in P.R. China, with the 11^th ^Five Year Plan (2006-2010) having introduced the "Building a Socialist New Countryside" campaign [46]. In order to monitor and evaluate this campaign, however, it is crucial to have a time- and cost-effective appraisal of relative SEP [47]. This paper supports the use of the asset-based index as a proxy measure of wealth, with weights derived from either PCA or PAF, although we recommend caution when comparing aggregated data from various settings. Given the renewed interest in the role of inequalities on economic inefficiency [48], and the important role of P.R. China in achieving the Millennium Development Goals (MDGs) [49], it is conceivable that these methods will be of use in numerous other applications, as well as in other geographical locations.

## Competing interests

The authors declare that they have no competing interests.

## Authors' contributions

JB was involved in the study design and in the implementation and co-ordination of the fieldwork, analyzed the data and drafted the manuscript. DPM, YSL and JU contributed to the study conception and design. ZYZ and LPY oversaw the fieldwork implementation. GMW assisted with the statistical analysis. YL, MYR, ZCL and JZ ran the household surveys in Wuyi and Laogang. GR supervised JB, contributed to the study conception, design, and implementation in the field. All authors were involved in critical revision of the manuscript and read and approved the final manuscript.

## Appendix Definitions of economics and statistics terms used in this paper

### Anti-image correlation matrix

A matrix containing the negatives of the partial correlation coefficients. Most of the off-diagonal elements should be small in a good factor model.

### Asset

An item of ownership convertible into cash.

### Bartlett's test of sphericity

A method to test whether the correlation matrix is an identity matrix, which would indicate that the factor model is inappropriate.

### Cronbach's coefficient α

A method of assessing the internal consistency, or reliability, of a set of items, where [(1-α^2^) × 100] indicates the percent of variance in the items that could be attributed to measurement error.

### Durables

Manufactured products such as an automobile or a household appliance that can be used over a relatively long period without being depleted or consumed.

### Eigenvalue

The scalar of the associated eigenvector, indicating the amount of variance explained by each PC or each PF.

### Eigenvector

A vector that results in a scalar multiple of itself when multiplied by a matrix. It corresponds to the weights in a linear transformation when computing PCA and PAF.

### Kaiser-Meyer-Olkin (KMO)

A measure of sampling adequacy which tests whether the partial correlations among items are small.

### Kruskal-Wallis

A non-parametric method for testing equality of population medians among groups.

### Peri-urban

Immediately adjoining an urban area; between the suburbs and the countryside.

### Principal axis factoring (PAF)

A data reduction technique which uses squared multiple correlations as initial estimates of the communalities. The communalities are entered into the diagonals of the correlation matrix before factors are extracted from the matrix, allowing the variance of each item to be a function of both item communality and non-zero unique item variance.

### Principal components analysis (PCA)

A data reduction technique using the principle components model. It assumes that components are uncorrelated and that the communality of each item sums to one for all components, therefore implying that each item has zero unique variance.

### Rotation

Turning the reference axes of the factors about their origin in order to achieve a simpler and theoretically more meaningful factor solution than is produced by the unrotated factor solution; the positions of the items are fixed in geometric space while the factor axes are rotated through specified angles.

### Socio-economic position (SEP)

An aggregate concept that includes both resource-based and prestige-based measures. Resource-based measures refer to material and social resources and assets, including income, wealth, and educational credentials. Prestige-based measures refer to individuals rank or status in a social hierarchy, typically evaluated with reference to peoples' access to, and consumption of, goods, services and knowledge, as linked to their occupational prestige, income, and educational level.

### Utility

A public service such as plumbing, electricity or railroad line.

## Supplementary Material

Additional file 1 **Step-by-step procedure for generation of asset-based wealth indices**. A step-by-step procedure for the generation of asset-based wealth indices, as used in this study. Click here for file
