# Supplementary material for: Comparison of two approaches for measuring household wealth via an asset-based index in rural and peri-urban settings of Hunan province, China
Source: Emerg Themes Epidemiol. 2010 Sep 3;7:7. doi: 10.1186/1742-7622-7-7 (PMC2942820; doi:10.1186/1742-7622-7-7)
Supplement: Additional file 1 — Step-by-step procedure for generation of asset-based wealth indices. A step-by-step procedure for the generation of asset-based wealth indices, as used in this study. [file 1742-7622-7-7-S1.DOC]

**Additional File Step-by-step procedure for generation of asset-based wealth indices**

1. Organise binary data into matrix with *m* households (rows) and *n* variables (columns).
2. Examine matrix for internal consistency.
3. Remove multicollinear variables.
4. Determine matrix factorability, using Bartlett’s test of sphericity and the Kaiser-Meyer-Olkin (KMO) test.
5. Exclude variables in a stepwise manner until a factorable *m* by *n* correlation matrix is reached.
6. Test for sampling adequacy by checking off-diagonal values of the anti-image correlation matrix.
7. Extract principal components (PCs) or principal factors (PFs) from final matrix.
8. Rotate factors.
9. Select best rotation (or no rotation) according to the maximum squared factor loadings and the relative simplicity of the model.
10. Examine scree plot, (a plot of extracted factors against their eigenvalues in descending order of magnitude).
11. Examine cumulative proportion of variance explained by each PC or PF with eigenvalue >1.
12. Identify variables loading with eigenvector > |0.3|.
13. If variables load equally on more than one PC or PF, use the Cronbach’s coefficient α  to select the PC or PF on which to place the variable.
14. Use principal (first) component or principal (first) factor to compute standardised indices of relative household wealth, according to the following equation:


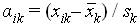

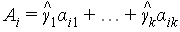


where


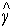
and *A_i_* is the standardised asset index score per household *i*,

the *_k_*’s are the factor loadings or weights of each asset *k*, and

the *α_ik_*’s are the standardised values of asset *k* for household *i*


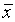
(i.e., *x_ik_* is the ownership of asset *k* by household *i*, 0 = not owning the asset,

1 = owning the asset, and *_k_* and *s_k_* = sample mean and standard deviation (SD), respectively, of asset *k* for all households).
